# Investigating the Impact of Nutrition and Oxidative Stress on Attention Deficit Hyperactivity Disorder

**DOI:** 10.3390/nu16183113

**Published:** 2024-09-15

**Authors:** Malina Visternicu, Viorica Rarinca, Vasile Burlui, Gabriela Halitchi, Alin Ciobică, Ana-Maria Singeap, Romeo Dobrin, Ioannis Mavroudis, Anca Trifan

**Affiliations:** 1 Faculty of Biology, “Alexandru Ioan Cuza” University of Iași, Carol I Avenue, No. 20A, 700505 Iași, Romania; malina.visternicu@yahoo.ro; 2 “Ioan Haulica” Institute, Apollonia University, Pacurari Street 11, 700511 Iași, Romania; rarinca_viorica@yahoo.com (V.R.); secretariat@univapollonia.ro (V.B.); alin.ciobica@uaic.ro (A.C.); 3 Department of Biology, Faculty of Biology, “Alexandru Ioan Cuza” University of Iași, Carol I Avenue, No. 20A, 700505 Iași, Romania; 4 Doctoral School of Geosciences, Faculty of Geography and Geology, “Alexandru Ioan Cuza” University of Iași, Carol I Avenue, No. 20A, 700505 Iași, Romania; 5 CENEMED Platform for Interdisciplinary Research, “Grigore T. Popa” University of Medicine and Pharmacy of Iasi, University Street No. 16, 700115 Iași, Romania; 6 Academy of Romanian Scientists, No. 54, Independence Street, Sector 5, 050094 Bucharest, Romania; 7 Department of Gastroenterology, “Grigore T. Popa” University of Medicine and Pharmacy of Iasi, University Street No. 16, 700115 Iași, Romania; ancatrifan@yahoo.com; 8 Institute of Gastroenterology and Hepatology, “St. Spiridon” University Hospital, 700115 Iași, Romania; 9 Institute of Psychiatry “Socola”, 36 Bucium Street, 700282 Iași, Romania; romeodobrin2002@gmail.com; 10 Department of Psychiatry, Faculty of Medicine, “Grigore T. Popa” University of Medicine and Pharmacy of Iasi, University Street No. 16, 700115 Iași, Romania; 11 Department of Neurology, Leeds Teaching Hospitals, NHS Trust, Leeds LS2 9JT, UK; mavroudis@nhs.net; 12 Faculty of Medicine, Leeds University, Leeds LS2 9JT, UK

**Keywords:** ADHD, oxidative stress, diet, gut–brain axis, gut microbiota

## Abstract

**Background/Objectives:** Attention deficit hyperactivity disorder (ADHD) is the most common childhood-onset neurodevelopmental disorder, characterized by difficulty maintaining attention, impulsivity, and hyperactivity. While the cause of this disorder is still unclear, recent studies have stated that heredity is important in the development of ADHD. This is linked to a few comorbidities, including depression, criminal behavior, and anxiety. Although genetic factors influence ADHD symptoms, there are also non-genetic factors, one of which is oxidative stress (OS), which plays a role in the pathogenesis and symptoms of ADHD. This review aims to explore the role of OS in ADHD and its connection to antioxidant enzyme levels, as well as the gut–brain axis (GBA), focusing on diet and its influence on ADHD symptoms, particularly in adults with comorbid conditions. **Methods**: The literature search included the main available databases (e.g., Science Direct, PubMed, and Google Scholar). Articles in the English language were taken into consideration and our screening was conducted based on several words such as “ADHD”, “oxidative stress”, “diet”, “gut–brain axis”, and “gut microbiota.” The review focused on studies examining the link between oxidative stress and ADHD, the role of the gut–brain axis, and the potential impact of dietary interventions. **Results**: Oxidative stress plays a critical role in the development and manifestation of ADHD symptoms. Studies have shown that individuals with ADHD exhibit reduced levels of key antioxidant enzymes, including glutathione peroxidase (GPx), catalase (CAT), and superoxide dismutase (SOD), as well as a diminished total antioxidant status (TOS) compared to healthy controls. Additionally, there is evidence of a close bidirectional interaction between the nervous system and gut microbiota, mediated by the gut–brain axis. This relationship suggests that dietary interventions targeting gut health may influence ADHD symptoms and related comorbidities. **Conclusions**: Oxidative stress and the gut–brain axis are key factors in the pathogenesis of ADHD, particularly in adults with comorbid conditions. A better understanding of these mechanisms could lead to more targeted treatments, including dietary interventions, to mitigate ADHD symptoms. Further research is required to explore the therapeutic potential of modulating oxidative stress and gut microbiota in the management of ADHD.

## 1. Introduction

Attention deficit hyperactivity disorder is one of the most common neuropsychiatric disorders affecting up to 4% of adults. ADHD is often characterized by severe symptoms during childhood that may persist and evolve with advancing age [1]. ADHD is associated with an increased risk of other psychiatric disorders, addictions, and accidents [2], prompting researchers to analyze the various risk factors involved in its pathogenesis [2,3]. Both psychostimulant and non-psychostimulant drugs are used to treat ADHD, with psychostimulant drugs showing greater efficacy over a shorter period [4].

OS induced by reactive oxygen species (ROS) and reactive nitrogen species adversely affects neuronal function and contributes to the pathogenesis of ADHD. In this context, the gut microbiota plays a crucial role in modulating OS and systemic inflammation, thereby directly impacting mental health. Dietary patterns are instrumental in regulating the microbiota and, consequently, in affecting neural function and the expression of psychiatric symptoms. Diets rich in antioxidants can mitigate OS, while foods that support microbiota health may help improve the symptoms associated with psychiatric disorders [5].

Recent studies have increasingly focused on the contribution of OS to the pathophysiology of ADHD [6,7]. OS is a biological condition characterized by an imbalance between oxidants and antioxidants, leading to excessive production of oxidants or insufficient antioxidant defenses [8]. This imbalance can cause damage to lipids, proteins, and DNA; alter cellular signaling and gene expression; inhibit protein activity; and induce apoptosis [9]. ROS, which include free radicals and non-radical compounds derived from oxygen, play a critical role in this process by degrading carbohydrates, proteins, lipids, and nucleic acids [9,10]. While low levels of ROS are necessary for normal cellular function, excessive ROS levels can lead to significant cellular damage [11].

The body’s defense against OS includes various antioxidants, some of which can cross the blood-brain barrier and protect nerve cells. Key antioxidant enzymes, such as catalase (CAT), superoxide dismutase (SOD), and glutathione peroxidase (GPx) are recognized biomarkers of oxidative stress [11]. Current research also underscores the importance of diet in managing ADHD, particularly through the intake of specific micronutrients like vitamins B and D and minerals such as manganese (Mn), zinc (Zn), magnesium (Mg), copper (Cu), selenium (Se), iron (Fe), and polyunsaturated fatty acids (PUFA) [12,13]. For example, iron deficiency has been implicated in the pathophysiology of ADHD due to iron’s essential role as a cofactor of tyrosine hydroxylase in catecholamine synthesis and metabolism, as well as in oxygen transport and brain myelination [14]. However, while supplementation with certain micronutrients can significantly reduce ADHD symptoms, excessive intake may lead to adverse effects. For instance, high doses of vitamin E supplementation have been linked to an increased risk of prostate carcinoma, and long-term ω-3 PUFAs (ω-3 polyunsaturated fatty acids) supplementation may be associated with serious side effects [12].

Additionally, the gut microbiome has emerged as a significant factor in ADHD, with its composition and function also being associated with other mental disorders. The gut–brain axis facilitates bidirectional communication between the gut microbiome and the brain [12,15]. The gut microbiota produces metabolites, such as serotonin, that influence neuronal function, while the brain sends signals, including dopamine, that can directly or indirectly impact the gut microbiota [16]. Disturbances in the intestinal microbiome are correlated with several conditions, including irritable bowel syndrome, cardiovascular diseases, immune disorders, obesity, and inflammatory bowel disease [16,17,18]. Without a healthy microbiome, pathogenic bacteria like *Clostridium difficile* increase, leading to gastrointestinal disorders such as diarrhea [19].

In this review, we will focus on the function of oxidative stress as a factor in the pathophysiology of ADHD, examine the influence of diet on treatment, and discuss the connection to the gut microbiome in the context of ADHD.

## 2. Methodology

### 2.1. Search Strategy

The current systematic review was conducted following the Preferred Reporting Items for Systematic Review and Meta-Analysis (PRISMA) guidelines, employing several electronic databases (Science Direct, PubMed, and Google Scholar) to conduct a comprehensive and systematic search using the following search terms: ((ADHD [Title/Abstract]) AND (diet [Title/Abstract])) AND ((ADHD [Title/Abstract]) AND (oxidative stress [Title/Abstract])) AND ((ADHD [Title/Abstract]) AND (gut–brain axis [Title/Abstract])) AND ((ADHD [Title/Abstract]) AND (gut microbiota [Title/Abstract])). The inclusion criteria focused on studies published up to July 2024 in English, which evaluate the relevance of oxidative stress biomarkers from different biological samples and their levels compared to control groups, the possible effects of various micronutrients on oxidative stress and brain function, as well as the role of diet in ADHD, with a particular focus on micronutrient supplementation.

### 2.2. Excluding Criteria

We applied the following exclusion criteria: (1) case reports, letters, summaries, expert opinions, and comments; (2) conference abstracts, books, book chapters, and unpublished results; and (3) non-English papers.

### 2.3. Data Extraction

Out of the initial 4482 reports collected through electronic search, 1370 were omitted due to duplication, 74 were excluded based on article type, and an additional 1810 were excluded as they comprised conference abstracts, books, book chapters, and unpublished results. Furthermore, 53 were excluded because they were not in English.

### 2.4. Data Synthesis

**Figure 1:**
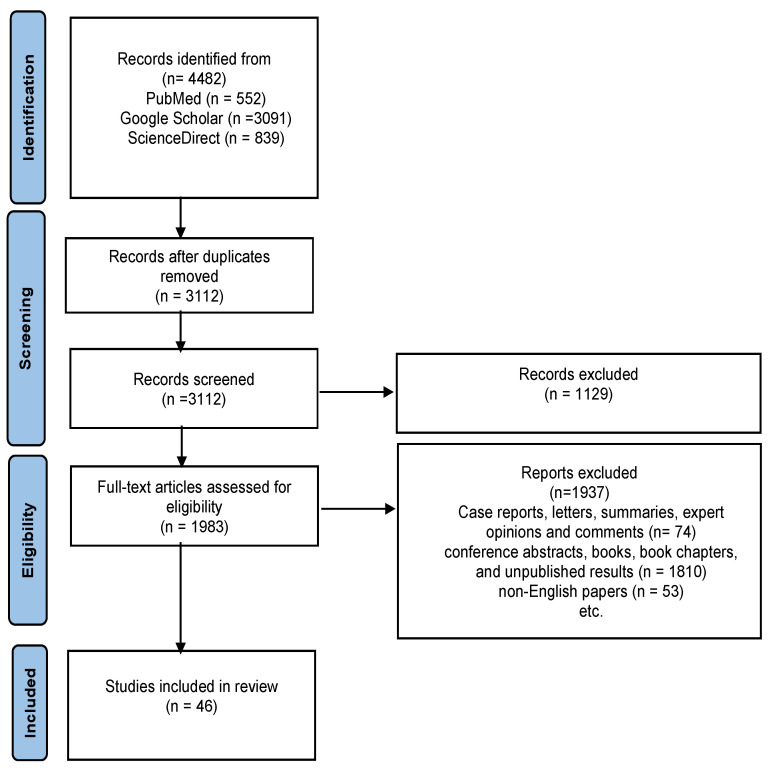
PRISMA flow chart illustrating the selection of studies and exclusion criteria.

Finally, 46 articles were included in this study, as shown in the diagram of the literature search and selection process (Figure 1). Due to the heterogeneity of the studies, narrative synthesis was deemed the most suitable approach. The results are summarized in three tables that address oxidative stress biomarkers in ADHD, different types of micronutrients, and their possible effects, with a focus on oxidative stress and micronutrient supplementation in individuals with ADHD.

## 3. ADHD

Attention deficit hyperactivity disorder is a prevalent neuropsychiatric disorder that is diagnosed based on persistent developmental levels of hyperactivity, inattention, and impulsivity [20]. ADHD is classified into three subtypes: inattentive, hyperactive-impulsive, and combined. Individuals with the inattentive subtype are often described as daydreaming, struggling to maintain focus, easily distracted, and less organized. The hyperactive-impulsive form is characterized by distractibility, reduced persistence, and lack of impulse control. The combined subtype presents with symptoms in all three categories. Additionally, there are gender differences in the subtypes: boys are more frequently diagnosed with the hyperactive-impulsive, whereas girls are more commonly diagnosed with the inattentive subtype [21]. In adults, hyperactivity often manifests as feelings of inner restlessness or agitation, incessant mental activity, excessive talking, and an inability to relax [3]. Impulsive behavior and interpersonal conflicts are correlated with negative consequences on relationships with friends, family, and colleagues. More specifically, impulsive behavior may often manifest as a coping mechanism to combat anxiety or as a result of a need for immediate gratification. Inattentiveness is characterized by distraction and slow processing speed. However, some patients, particularly those engaged in activities of interest, may exhibit hyperfocus or overconcentration [3]. ADHD frequently co-occurs with other psychiatric disorders, such as depression, anxiety, bipolar disorder, autism spectrum disorders, conduct disorder, eating disorders, and substance use disorders [22]. ADHD has a high degree of heritability, estimated at approximately 76%. Furthermore, prenatal risk factors such as alcohol and drug use, as well as hypertension, play a significant role in the pathogenesis of ADHD [3]. Moreover, maternal stress during pregnancy and premature birth are environmental factors associated with ADHD [3].

The etiology of ADHD is considered multifactorial, with genetic predisposition and biological vulnerability being the most significant contributors. Comorbidity is common among children with ADHD, with conduct disorders, depression, and anxiety being the most frequently observed conditions observed in children with ADHD [23,24]. Other disorders, such as autism spectrum disorder and exposure to environmental agents and substances, including lead (Pb), alcohol, tobacco, and other drugs, may also contribute to the symptoms and pathogenesis of ADHD [21]. While understanding the onset and progression of ADHD is important [23], no single cause has been identified, and exposure to a risk factor does not guarantee the development of ADHD. The factors contributing to the onset of ADHD may differ from those influencing its progression and outcomes. Genetic factors may exert indirect effects through interactions with environmental factors [25]. Prenatal and postnatal factors such as low birth weight, premature birth, and maternal substance use during pregnancy have been linked to an increased risk of developing ADHD [23].

### 3.1. Factors Influencing ADHD Symptoms

ADHD symptoms are influenced by a wide range of factors, reflecting the complex and multifactorial nature of the disorder. Accurately identifying specific environmental risk factors for ADHD is challenging, as the observed associations may either be consequences of ADHD symptoms or may result from other disorders in the child or parents. Although the number of ADHD diagnoses has increased, studies have not indicated a significant rise in the prevalence of ADHD in the population over time. This suggests that the environmental risk factors for ADHD are likely numerous, have small individual effects, and remain relatively stable across different regions and periods [25]. Several studies have indicated that early parent-child relationships can influence the manifestation of ADHD symptoms [24]. Additionally, a chaotic home environment may partially reflect the genetic risk of children with ADHD, contributing to the intensification of the disorder’s core symptoms [26]. Sleep disorders are common and persistent in children with ADHD, and diet also impacts symptomatology. However, the interactions between diet, sleep, and behavior in children with ADHD remain insufficiently studied [27].

Excessive maternal alcohol consumption during pregnancy is a known risk factor for fetal alcohol syndrome, which is associated with behavioral symptoms such as inattention and hyperactivity [25]. Maternal stress during pregnancy has also been linked to the development of ADHD symptoms in offspring. However, recent research suggests that this association may be more attributable to genetic inheritance between mother and child rather than a direct causal relationship [25]. Some studies indicate that premature birth or low birth weight may increase the risk of developing ADHD, with children born prematurely showing a significantly higher likelihood of developing the disorder compared to those born at term. Additionally, various dietary components have been examined for ADHD symptoms, including artificial colorants and sugar, Zn, Fe, Mg, and ω-3 fatty acids [25].

### 3.2. Factors Influencing the Pathogenesis of ADHD

Various factors contribute to the pathogenesis of ADHD, encompassing both genetic and environmental influences. Parental mental health issues, including ADHD, are significant risk factors for the development of psychopathology in children. Specifically, parental ADHD symptoms and maternal depression have been linked to the onset of ADHD in childhood. Additional familial risk factors include family conflicts and unfavorable parenting conditions, particularly inadequate emotional support for the child [23]. Environmental factors impacting ADHD include exposure to nicotine, polychlorinated biphenyls, ethanol, lead, radiation, 6-hydroxydopamine, ionizing substances, neonatal hypoxia, certain pesticides, and organic pollutants [21,25]. These substances can adversely affect the cognitive and neural systems involved in ADHD [25]. Early environmental factors, including those contributing to increased inflammation, such as maternal infections, obesity, nutritional deficiencies, viral infections, and maternal exposure to pollutants, are also relevant to the etiology of ADHD [6,28]. Environmental factors, particularly those encountered during early developmental stages, are well-documented to influence gene expression through epigenetic mechanisms, thereby affecting neuronal development [28].

Individuals with a genetic predisposition to ADHD are more susceptible to developing the disorder when exposed to certain environmental factors, many of which affect genes that regulate dopamine and serotonin receptors [28]. One of the strongest associations with ADHD is a variant of the dopamine receptor gene DRD4, which interacts with both dopamine and norepinephrine and contains a functional polymorphism in exon III. The dopamine receptor gene DRD5 has also been consistently linked to ADHD [25]. Family studies have shown that ADHD is more prevalent among parents and siblings of affected individuals compared to unaffected relatives. Twin studies indicate much higher concordance rates for ADHD in monozygotic twins than in dizygotic twins [25].

OS and energy metabolism are crucial factors in the pathology of ADHD [29]. Research has indicated that ADHD is frequently associated with elevated OS levels [6,30]. The pathophysiology of ADHD involves OS and neuroinflammation resulting from an imbalance between oxidants and antioxidants, catecholaminergic dysfunction, treatment-related effects, and environmental and genetic influences. In the brain, OS can compromise neuronal integrity due to the high concentration of PUFAs, which are highly susceptible to oxidation and can generate ROS, leading to oxidative neuronal damage [6]. Additionally, iron plays a significant role in this process, as it catalyzes reactions that generate oxidative stress. ADHD has been linked to low serum ferritin levels, with some individuals exhibiting iron deficiencies [30].

ADHD has a prevalence of 5% in children between the ages of 4 and 17 years, and many diagnosed children experience educational challenges or other mental health issues during adolescence [31]. In 2022, the prevalence of ADHD in children and adolescents was reported to be approximately 7% [13]. Children with ADHD show a higher frequency of learning and language, motor, cognitive, and mental health disorders. However, the symptoms and signs of ADHD can vary across different developmental stages of childhood [32]. Children with ADHD who do not receive treatment are at greater risk of dropping out of school and engaging in substance abuse [33]. Additionally, individuals with ADHD are more likely to experience mental health difficulties in adulthood, including anxiety disorders, mood disorders, and personality disorders [33].

Although ADHD is typically diagnosed in childhood, it persists into adulthood [34], with a prevalence of 3.2% in adult women. Interestingly, women with ADHD who do not exhibit symptoms in childhood often discover their condition after one of their children is diagnosed with the disorder. Women are twice as likely to have ADHD as men [35].

**Figure 2:**
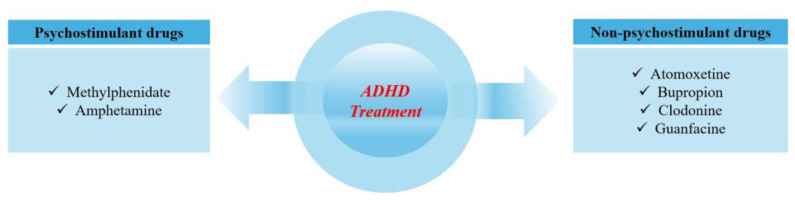
Treatment used for ADHD.

While the exact causes of ADHD are not fully understood, the disorder is commonly treated with psychostimulants [35,36], such as methylphenidate (MPH), dexmethylphenidate, dextroamphetamine, mixed amphetamine salts, pemoline, and modafinil [35]. Additionally, non-psychostimulant drugs such as atomoxetine, guanfacine, and bupropion are also used to treat ADHD [35,37]. Studies have identified that ADHD medication is associated with a 32% reduction in the risk of criminal behavior in men and a 41% reduction in women, with no significant differences between stimulants and atomoxetine [38]. Moreover, ADHD medications have been linked to a 20% decrease in unplanned hospital visits due to depression [39]. The most commonly used psychostimulant drugs for ADHD are MPH and amphetamine (Figure 2), which improve symptoms by blocking presynaptic dopamine and norepinephrine transporters and increasing catecholaminergic transmission in the striatum, prefrontal cortex, and hippocampus. This mechanism enhances neurotransmitter levels, which are essential for improving attention and minimizing impulsivity in individuals with ADHD [40]. Psychostimulants like MPH and amphetamines function by either inhibiting reuptake or increasing the release of these neurotransmitters [41]. Specifically, MPH blocks the reuptake of dopamine and norepinephrine by binding to and inhibiting their transporters [42], which raises the concentrations of these neurotransmitters in the synaptic cleft and boosts dopaminergic and noradrenergic activity in brain areas such as the prefrontal cortex, which play a critical role in attention and impulse control.

Additionally, amphetamines not only inhibit reuptake but also stimulate the release of dopamine and norepinephrine from presynaptic neurons [43]. This combined effect significantly enhances the availability of these neurotransmitters, leading to improved concentrations and a reduction in hyperactive behavior. On the other hand, non-psychostimulant medications, such as atomoxetine, operate differently. Atomoxetine selectively inhibits the reuptake of norepinephrine, which can increase attention and decrease impulsivity without the side effects commonly associated with psychostimulants [44]. Medications such as clonidine and guanfacine target alpha-2 adrenergic receptors in the brain [45], helping regulate neurotransmitter release and alleviating ADHD symptoms by reducing hyperactivity and impulsivity through their calming effects on the central nervous system.

In summary, both psychostimulant and non-psychostimulant ADHD treatments ultimately function by modulating the levels and activity of dopamine and norepinephrine in the brain, particularly in areas associated with attention, impulse control, and hyperactivity [40]. For non-psychostimulant treatment, this includes atomoxetine, which inhibits the transport of norepinephrine [6].

In adults, ADHD treatment aims to alleviate symptoms and manage comorbidities. The treatment approach for adults differs from that for children. For instance, in France, MPH is the only psychostimulant approved for treating this disorder, but it is not authorized for adult use [1]. Although treatment is associated with short-term side effects, such as decreased appetite [46], weight loss, headaches, anxiety [13,46], and sleep problems [47], it also has long-term effects, such as impacting bone health [46,48]. MPH can lead to induced oxidative stress [49,50] by increasing reactive nitrogen and oxygen species that can alter antioxidant defense mechanisms, potentially involving enzymes, which consequently links to lipid, protein, and DNA damage [50,51,52] MPH can cause oxidative damage in the prefrontal cortex by promoting the auto-oxidation of catecholamines, such as dopamine and norepinephrine. This process generates reactive oxygen species, which in turn leads to oxidative damage and, potentially, cell death. Similarly, atomoxetine treatment increases extracellular catecholamine levels and ROS, which may contribute to the pathophysiology of ADHD [6]. In contrast, guanfacine and clonidine are associated with side effects like sedation, drowsiness, and abdominal pain. Despite these side effects, the risk of major cardiovascular events associated with these medications remains low [13].

Recent studies have highlighted the therapeutic benefits of probiotics and prebiotics in managing psychiatric disorders, including ADHD [5,13]. Probiotics are live microorganisms found in fermented foods and nutritional supplements [53]. They exert beneficial effects through different mechanisms, such as lowering the intestinal pH, decreasing the invasion and colonization of pathogenic organisms, and modifying the host immune response [53]. Research on animal models of depression and stress has shown that probiotic treatment can alter the gut microbiota composition and enhance the peripheral levels of the precursor serotonin and tryptophan [53,54]. Moreover, probiotics contribute to regulating the immune system, restoring intestinal balance, and supporting the integrity of the intestinal barrier [5]. Psychological therapies and dietary interventions have also been employed to manage ADHD, complementing pharmacological treatments. Among these, behavioral therapy has been proven to be the most effective. Although healthy diets such as the Mediterranean diet show promise in reducing the risk of ADHD, evidence supporting non-pharmacological treatments remains limited and requires further investigation. In contrast, unhealthy eating habits are associated with a higher risk of ADHD, whereas diets rich in fruits and vegetables are linked to a lower risk [13].

## 4. Oxidative Stress in ADHD

In recent years, more studies have reported the influence that OS has on neuropsychiatric disorders, but its mode of action is still unclear [10]. Oxidative stress occurs when there is an imbalance characterized by the overproduction of oxidants, reduction in antioxidant levels, or both. This imbalance leads to harmful effects, as excess free radicals or a compromised antioxidant system disrupts central nervous system enzymes and alters neurotransmitter receptor functions [8]. Higher levels of OS have been observed in people with ADHD, particularly in children with this disorder, where higher levels of malondialdehyde (MDA) have been recorded compared to controls [6]. In numerous randomized, double-blind, placebo-controlled trials, participants with ADHD were found to have a lower total oxidant status (TOS) [55,56].

Key antioxidants associated with ADHD include γ-linolenic acid (GLA) [57], total antioxidant capacity (TAC) [58], antioxidant glutathione (GSH) [9], melatonin [59], and total antioxidant status (TAS) [56]. Additionally, several important enzymes work as markers of OS, including glutathione peroxidase [60], catalase (CAT) [61], superoxide dismutase (SOD) [62], and glutathione-S-transferase (GST) [63]. Oxidants such as lipid peroxidation products (LPO) [61], plasma levels of advanced oxidation protein product (AOPP) [61], nitrites + nitrates (NOx) [61], glutathione reductase activity (GRd) [61], total thiols [8], MDA [64] and TOS [56] are also critical in understanding OS in ADHD.

GPx is an antioxidant enzyme critical for combating OS and maintaining redox balance [65]. A study conducted on 35 ADHD patients from Gazi University Faculty of Medicine, Child, and Adolescent Psychiatry Clinic and 35 healthy control patients reported significantly lower plasma GPx level ADHD patients compared to the healthy group [60]. Another important enzyme, CAT, plays an essential role in the metabolism of H_2_O_2_ and reactive nitrogen species [66]. In this context, although CAT enzyme activity in plasma was higher compared to that in the controls, the difference was not statistically significant [60]. CAT activity in saliva was estimated in ADHD patients and controls and was found to be reduced in patients with ADHD compared to healthy controls [67]. Moreover, the mean serum TAC, GSH, and CAT levels of patients with ADHD were significantly lower than those of the healthy group [58]. However, decreases in plasma TAS levels were observed in children diagnosed with ADHD compared to the reference values in control patients [68,69].

In serum, MDA levels have been reported to be increased [64], although some studies have indicated statistically significantly lower values [70]. There is also evidence showing a significant decrease in TAS in one study [55], while other research indicates lower levels, but without reaching statistical significance [68,69]. At the same time, higher levels of melatonin have been observed [59].

Both SOD1 and TAC (Table 1) showed significantly lower values in the serum [58,68], while in the plasma, total thiols and GST also showed significant decreases compared to those in the control group [8,63]. Garre-Morata et al. 2024 found that after administration of MPH for three months, plasma levels of AOPP, LPO, and NOx decreased [61]. In addition, studies examining the effects of MPH on the rat brain reported a decrease in the activity of antioxidant enzymes, such as CAT and SOD [71,72,73].

## 5. Food Diet in ADHD

Diet can affect the gut microbiome, which in turn influences a variety of physiological processes, including the immune system, appetite, metabolism, nutrient absorption, and neuronal development via GBA [76]. Additionally, high-calorie foods combined with a lack of exercise contribute to the damage of fat cells, leading to significant health problems such as obesity [77]. Ingested food causes the gastrointestinal tract to respond by signaling the brain to induce satiety [77]. The intestinal epithelium, consisting of absorptive enterocytes, proliferative stem cells, and secretory cells, including enteroendocrine cells, is the primary interface between the host and the microbiota. Enteroendocrine cells, which make up 1% of the intestinal epithelium, represent the largest network of endocrine cells in the body that express numerous hormones [78]. In response to food ingestion, L cells secrete hormones [77], such as glucagon-like peptide-1 (GLP-1) and peptide YY (PYY). L cells are sensitive to short-chain fatty acids (SCFA) and secondary bile acids, but they respond most intensely to microbiota-derived nutrients and metabolites, stimulating hormone secretion [78]. GLP-1 has multiple effects, including insulin secretion and increased satiety, while PYY reduces food intake [78].

Food nutrients are known to have powerful antioxidant and anti-inflammatory properties for maintaining cellular redox homeostasis by activating antioxidant defense systems such as the nuclear factor erythroid 2–related factor 2 (Nrf2) pathway and the phase II detoxification genes and enzymes including heme oxygenase-1 (HO-1), heat shock protein 70 (Hsp70), sirtuin-1 (Sirt1), GPx, thioredoxin (Trx), SOD, and CAT for neuroprotection during OS and neurotoxicity that lead to the onset and progression of neuropsychiatric disorders such as ADHD [79,80,81].

It is interesting to note that nutrients, particularly polyphenols, vitamins, probiotics, and PUFAs, follow the concept of hormesis, a biphasic dose-response process by which small, nontoxic stresses or mild stress are used to induce cellular adaptive responses that protect the biological system against subsequently large and potentially lethal stresses of the same, similar, or different nature. According to hormesis, low doses of food nutrients are neuroprotective by activating Nrf2 antioxidant pathways, while high doses can be toxic and induce inhibition of these protective pathways, causing brain damage. Therefore, polyphenols, but also probiotics and PUFAs, can be considered as “hormetic nutrients” since they are capable of orchestrating cellular resilience to stress and the preservation of cellular redox homeostasis as benchmarks of health by activating intracellular antioxidant enzymes such as SOD, CAT, and GPx in a dose-dependent manner. This is in line with emerging evidence [82,83,84] that shows that a low dose of hormetic nutrients upregulates antioxidant Nrf2 pathways for enhancing the stress resilience response to counteract excess free radicals in vitro and in vivo. On the other hand, high doses of natural compounds can be toxic to cells and animal models, leading to the inhibition of antioxidant pathways and the onset and progression of neurological disorders associated with OS. The field of hormetic/adaptive responses activated by food nutrients in enhancing endogenous redox defense systems and reducing OS is emerging as a promising preventive and therapeutic approach in gut–brain axis disorders [81]. In this context, the interaction between polyphenols and probiotics plays a significant role, with the potential to prevent and treat inflammation associated with changes in the intestinal microbiota, positively influencing the gut–brain axis. For instance, resveratrol, a natural polyphenol found in grape skins, not only provides antioxidant and anti-inflammatory effects, but also supports neuroprotective functions. By modulating the intestinal microbiota, resveratrol affects the GBA, contributing to the regulation of insulin sensitivity and mitochondrial biogenesis through the activation of Sirt1. Resveratrol also activates the Nrf2 pathway, offering protection against OS and reducing neuroinflammation [81]. Similarly, curcumin not only regulates the intestinal microbiota by promoting beneficial bacteria and reducing pathogenic ones but also activates the Nrf2 pathway, thereby protecting against OS and inflammation. Additionally, it influences the GBA, improving both intestinal symptoms and cognitive function by balancing neurotransmitters and modulating the microbiota [81,85].

Blueberries, which are rich in anthocyanins, fiber, and polyphenols, offer antioxidant and anti-inflammatory effects that support healthy intestinal microbiota and enhance brain function. They modulate the Nrf2 pathway, stimulate the production of antioxidant enzymes, and protect against OS. Regarding the gut–brain axis, blueberries improve microbiota composition by increasing beneficial bacteria such as Lactobacillus and Bifidobacterium, while reducing pathogenic bacteria [81]. Another example is Hidrox, an olive extract containing hydroxytyrosol, which activates the Nrf2 pathway to reduce OS and neuroinflammation. It improves the intestinal microbiota by increasing beneficial bacteria and supporting cognitive health [81]. Additionally, Hidrox has shown promise in reducing inflammation and managing Alzheimer ’s-like diseases [86]. Saffron also contributes to Nrf2 activation, reducing OS and inflammation in both the intestine and the brain. In the gut, saffron regulates the microbiota by decreasing pathogenic species and combating dysbiosis [81]. Moreover, polyphenols from the Hericium erinaceus mushroom stimulate the Nrf2 pathway, exhibiting similar effects in reducing OS and neuroinflammation. They enhance the intestinal microbiota by increasing beneficial bacteria and SCFAs, while protecting the brain by inhibiting amyloid-β accumulation, thereby improving both gut health and cognitive function [81,87].

The dose is a crucial determinant for inducing protective or harmful effects and should be carefully evaluated. Therefore, finding the optimal dose of dietary nutrients in order to promote healthy effects is crucial for achieving protection and/or toxicity. Numerous recent evidence [80,88,89,90] have shown that low doses of dietary nutrients promote neuroprotection via the activation of the Nrf2 pathway in order to prevent or attenuate OS and cognitive damage in vitro and in vivo.

In addition, dietary interventions can influence behavior and mental health, as supplementing or restricting certain nutrients or foods can result in positive effects on improving ADHD symptoms [91]. Nutrition plays an essential role in neuropsychiatric disorders, demonstrating that children with ADHD often present with micronutrient deficiencies [92]. Adequate vitamin and mineral intake is crucial for normal brain development, as deficiencies can impair the brain regions involved in the pathogenesis of ADHD [93]. Following a diagnosis of ADHD, the National Institute for Health and Care Excellence in the United Kingdom recommends a balanced diet and nutrition, along with regular exercise, as drug treatment is not recommended for preschoolers [94]. Additionally, to reduce ADHD symptoms, the Mediterranean diet is recommended [95], which involves a high consumption of unsaturated and monounsaturated fats and a high intake of vegetables, fruits, legumes, and cereals. Another approach is the elimination diet, which is based on the premise that adverse reactions to certain foods may be linked to ADHD and involves the elimination of food additives or products with a high degree of allergy [96].

Several studies have highlighted the potential role of micronutrients in neurodevelopmental disorders (Table 2) [97,98,99].

Mn is an essential element because it functions as a coenzyme in various biological processes [116]. It is involved in bone formation, macronutrient metabolism, and defense systems against free radicals [116] and plays a fundamental role in brain function and development [117]. Moreover, Mn acts as an activator of some antioxidants and is critical for maintaining insulin synthesis and secretion [118]. As a component of manganese superoxide dismutase (MnSOD), Mn is vital for scavenging reactive oxygen species during mitochondrial oxidative stress [118]. MnSOD is a primary antioxidant that scavenges superoxide in the mitochondria, providing protection against OS [118]. Major dietary sources of Mn include teas, juices, cereals, rice, nuts, seafood, chocolate, fruits, vegetables, seeds, and spices [100]. Mn deficiency is associated with numerous disorders, such as impaired carbohydrate metabolism and brain malfunction [119]. In addition, elevated Mn levels have been reported in children with cognitive deficits and attention and learning problems, influencing the dopaminergic system [120].

Mg is the most common cation with significant physiological importance [121] and is involved in the development and functioning of the brain [122]. It is found primarily in plant foods (vegetables, legumes, grains, seeds, and nuts), the consumption of which is recommended to reduce ADHD symptoms [123]. Mg deficiency can present with symptoms such as aggression, fatigue, nervousness, and attention deficits [124]. Magnesium supplementation has also been shown to reduce ADHD symptoms in children [121]. In a study involving 148 boys aged 4 to 9 years, including 44 with ADHD, 40 with autism spectrum disorder, and 32 with both ADHD and autism spectrum disorder, the analysis of blood serum, urine, and hair samples revealed that hair Mg levels were significantly lower in children with ADHD compared to controls [92].

Zn is an essential micronutrient involved in numerous metabolic processes, including the proper functioning of the immune system, protein synthesis, DNA synthesis, and cell division [125]. Zn has also been associated with ADHD [126] and plays a role in the production of melatonin, which is necessary for dopamine metabolism [127]. Since Zn is required for melatonin metabolism, it is considered an important factor in the treatment of ADHD [128]. The main food sources of Zn are poultry, red meat, seafood, nuts, and seeds. Consumption of these foods is recommended because Zn has antioxidant properties and can protect against OS [101,102,123]. Children with ADHD have been found to have much lower serum Zn levels [129]. In 2020, Robberecht et al. observed lower Zn levels in the hair and nails of children with ADHD [121]. Another study demonstrated that 6 to 10 weeks of Zn and Fe supplementation improved ADHD symptoms [125].

Iron is another important micronutrient that is essential in various biological processes, such as oxygen transport by hemoglobin [130]. Fe is vital for basic brain function [131]. As a cofactor of tyrosine hydroxylase, its deficiency leads to lower dopamine production, resulting in increased ADHD symptoms and affecting behavior [121]. Fe supplementation shows clinical utility in patients with ADHD [132]. However, when present in excess, it disrupts redox homeostasis and catalyzes the propagation of ROS, leading to OS [130]. In the etiology of ADHD, Fe deficiency is one of the basic nutritional factors with a role in the essential functions of the brain, including myelination, the development of oligodendrocytes, and the synthesis of neurotransmitters [133]. However, there are other micronutrients that have a role in reducing ADHD symptoms, among them Se, and Cu, the latter having a role in catecholamine metabolism. Selenium is found in foods such as vegetables, fish, and meat. It functions as a cofactor of GPx enzymes, which play a role in protecting against OS [106,107], and a high Cu/Zn ratio may contribute to the risk of ADHD [121].

Vitamins are organic compounds obtained primarily through diet, except for vitamin D, which is synthesized through sun exposure [134]. Besides sunlight, vitamin D can be sourced from foods such as fish, dairy products, cereals, orange juice, and eggs [49,112]. Vitamin D deficiency has been associated with neurodevelopmental disorders, including attention deficit hyperactivity disorder, and its deficiency affects the expression of synaptic proteins [135]. On the other hand, vitamin B6 is found in meat, dairy products, nuts, fruits and vegetables [109], and vitamin B12 in meat, milk, eggs and fish [108]. Lower levels of B vitamins (B2, B6, B9, B12) have been significantly associated with ADHD. Vitamin B12 facilitates the conversion of homocysteine to methionine via methionine synthase, an essential reaction in the nucleotide synthesis process [134]. Both vitamin B6, B12, and vitamin D play a role in reducing OS [49,108,109,110,111,112].

Deficiencies in certain unsaturated fatty acids may contribute to ADHD [136]. PUFA are essential for normal neurotransmitter function [137]. ω-3 PUFAs, found in fish, microalgae, and some microorganisms, play a role in reducing the ratio of SOD/CAT enzymes [113,114,115]. Supplementation with ω-3 fatty acids can improve ADHD symptoms [95], and a balanced ratio of ω-3 to ω-6 PUFA may be even more effective [138]. More specifically, fish oil supplementation may represent a promising dietary intervention, although the common addition of vitamin E to fish oil could raise concern [54].

Regarding dietary options for ADHD, supplementation with minerals, vitamins, and PUFA is common (Table 3) [13]. Because sugar, sweets, artificial food colors, and preservatives are associated with ADHD symptoms [139,140], some authors recommend eating fruits, vegetables, fish, and other foods high in PUFA and other micronutrients to protect against OS and reduce the risk of ADHD [13].

Numerous studies (Table 3) confirm that micronutrient supplementation can improve symptoms of attention deficit hyperactivity disorder. For instance, supplementation with 150 mg Zn for 12 weeks had a positive result [63], while administration of a smaller amount (15 mg) for 13 weeks did not have a positive effect, leading researchers to not recommend this dose [63]. In another study involving 52 patients aged 7–14 years, 0.5–1 mg/kg/day of Zn was administered for 6 weeks alongside MPH treatment, confirming the positive effect of Zn [129].

Another study involving 23 participants aged 5–8 years confirmed that 80 mg/day of ferrous sulfate for 12 weeks improved ADHD symptoms in children with low serum ferritin [143]. Furthermore, supplementation with 200 mg/day of Mg for 6 months showed a positive effect on ADHD symptoms [144]. In another study, 74 participants were supplemented for 8 weeks with 50,000 IU/week of vitamin D and 6 mg/kg/day of magnesium supplements, yielding positive results, as vitamin D and magnesium supplementation in children with ADHD reduced behavioral problems, social problems, and anxiety [147]. Vitamin D supplementation also improved ADHD symptoms [146,147], as observed in a study where 96 patients were given 50,000 IU/week 25-hydroxy-vitamin D3 for 6 weeks [145], as well as in a study involving 35 patients aged between 7 and 14 years who were administered 3000 IU/day 25-hydroxy-vitamin D3 over 12 weeks [146].

The efficacy of ω-3 PUFA was tested in a study involving 40 patients aged between 8–14 years, who were given 10 g daily of margarine enriched with either 650 mg of eicosapentaenoic acid (EPA)/docosahexaenoic acid (DHA), for 16 weeks, and the result was positive [148]. ω-3 PUFAs, particularly DHA and EPA, enhance cellular membrane fluidity, neurotransmission, and receptor function [149]. DHA deficiency is associated with dysfunctions in the dopaminergic system [150], but supplementation with ω-3 PUFAs has been shown to improve attention and reduce impulsivity. Additionally, these fatty acids exhibit anti-inflammatory effects, which contribute to reduced levels of proinflammatory interleukins [149].

## 6. ADHD vs. Gut−Brain Axis

In the last decade, researchers have focused on the connection between gut microbiota and neurodevelopmental disorders, revealing potential correlations [54]. Communication between the gastrointestinal tract and the brain is facilitated by the gut–brain axis, which includes the central nervous system, spinal cord, enteric nervous system, autonomic nervous system, and hypothalamic-pituitary-adrenal (HPA) axis [151]. The HPA axis is crucial for maintaining body homeostasis, and its regulation occurs mainly by changing cytokine secretion [5].

As stated by Gong et al. in 2023, the gut−brain-axis (GBA) links intestinal dysfunction and inflammation with brain function and neuropsychiatric disorder [152]. The interaction between GBA and gut microbiota is achieved through neural, immune, endocrine, and humoral links [153]. Furthermore, the mechanism underlying GBA communication involves neuro-immune-endocrine mediators. GBA plays the role of integrating gut function, linking the emotional and cognitive centers of the brain with gut function and mechanisms such as gut permeability, immune activation, entero-endocrine signaling, and enteric reflux [153].

The immune, neuroendocrine, and neural pathways each play distinct roles in modulating the interactions between the gut and the brain. The immune pathway is particularly crucial, with the gut microbiome being essential for maintaining and developing both the intestinal barrier and the blood-brain barrier. Alterations in the microbiome can impact the expression of tight junctions [154], potentially compromising these barriers to microorganisms and inducing neuroinflammation [149]. The microbiome influences the differentiation and maturation of immune cells [155], including microglia, which are involved in neurogenesis and the regulation of cognitive functions [149]. Disruption of the microbiome can affect microglial function [156]. Additionally, the microbiome impacts the recruitment of peripheral monocytes to the brain, mediated by tumor necrosis factor-alpha (TNF-α) and free fatty acid receptors, highlighting its role in regulating immune and neuroinflammatory responses. Furthermore, acquired immunity develops and matures in response to exposure to gut microbiota [149].

Another crucial pathway in the interaction between the gut microbiome and the central nervous system is the neuroendocrine pathway. The gut microbiome is integral to the development and functioning of the HPA axis, which regulates neuroendocrine responses and manages stress [149]. The neural pathway in the GBA is mediated by the vagus nerve, which transmits signals between the gut and the brain. The vagus nerve influences behavior and mood by modulating the brain’s response to mechanical and chemical stimuli from the gut. Such signals can influence the release of catecholamines in the brain, thereby influencing the states of depression and anxiety. Additionally, the gut microbiome, through its interactions with vagal afferents, can modulate emotional and behavioral responses, impacting anxiety-like and depressive behaviors. The enteric nervous system, which includes the myenteric and submucosal plexuses, coordinates intestinal functions, such as motility and fluid movement [149].

The intestinal microbiome has an increasingly recognized impact on the functioning of ADHD through its ability to synthesize neurochemical substances and precursors [157], with an important role in anti-inflammatory responses, helping to reduce psychiatric symptoms caused by inflammation [158]. Precursors of monoamines, such as dopamine, serotonin, and neuroadrenaline, are produced by several members of the gut microbiota [157]. In this context, there is a close connection between the gut–brain axis and neuropsychiatric disorders, such as ADHD. Gamma-aminobutyric acid (GABA), serotonin, dopamine, and norepinephrine are produced by the gut microbiota and influence GBA [159]. Serotonin is an important factor in the pathogenesis of ADHD because approximately 90% of serotonin is produced in the gut by enterochromaffin cells and the gut microbiota. Also, GABA is another factor that can contribute to ADHD pathology; the intestinal microbiota is able to influence the availability of GABA through the absorption and secretion of this neurotransmitter. Furthermore, bacterial metabolites such as SCFA increase the rate-limiting enzymes in the synthesis of noradrenaline, dopamine, and serotonin [76].

The gut microbiota is considered an environmental factor that regulates host metabolism by interacting with different tissues via microbiota-derived signals and metabolites [78]. The gut microbiota consists of six major phyla, Firmicutes, Bacteroides, Actinobacteria, Proteobacteria, Verrucobacteria, and Fusobacteria [160], which perform complex functions, including food and drug metabolism [54]. The gut microbiota has been associated with various aspects of human health, including metabolism, gut homeostasis, brain behavior, and immune development [54]. The gut microbiota has been found to play a particular role in health and neuropsychiatric disorders, as it can affect mental health and brain activity [161]. Gut microorganisms can impact central nervous system processes through immune system modulation and communication via the vagus nerve [54]. The vagus nerve is considered a vital channel for two-way communication between the brain and gut, which can differentiate between pathogenic and non-pathogenic bacteria [96]. Microorganisms that are part of the intestinal microbiota participate in the food digestion process by secreting digestive enzymes and transforming complex nutrients into simple organic compounds. Another role of the gut microbiota is the synthesis of vitamins, especially those in the B complex [5]. Through the GBA, diet can exert its effect on ADHD; this communication system between the enteric nervous system and the central nervous system includes the emotional and cognitive centers of the brain [151]. DNA sequencing can be used to investigate microbial composition and its role in the pathogenesis of ADHD [151].

Gut microorganisms produce various metabolites such as short-chain fatty acids, including butyrate, propionic acid, and acetate, through the breakdown of fibers and undigested sugars. These acids serve as essential energy sources for the mitochondria and play a crucial role in conditions involving inflammation, hunger, and intense physical activity [149]. SCFA are considered the main class of biological products of the intestinal microbiota that can be obtained by fermentation of dietary fibers. While the fermentation process of dietary fiber is essential in regulating gut microbiota composition, SCFA can affect host health at the cellular, tissue, and organ levels. Abnormalities in the production of these metabolites have been associated with ADHD symptoms [162].

Disruption of the gut microbiota caused by antibiotics, infections, poor diet, or stress can lead to dysbiosis [158]. This imbalance results in the growth of inflammatory microorganisms, which can affect intestinal permeability and causie systemic inflammation through microbial translocation. Systemic inflammation can disrupt the blood-brain barrier and elevate the levels of proinflammatory cytokines, contributing to oxidative stress and impacting neurotransmitters associated with ADHD [151]. However, further studies are needed to understand how the gut microbiota regulates proinflammatory cytokines, given the established link between the inflammatory process and pathogenesis of ADHD [151]. Moreover, the gut microbiome regulates the maturation and differentiation of macrophages, innate lymphoid cells, and dendritic cells. Macrophages constitute microglia [149], immune cells that represent 10% of the central nervous system [163], with an important role in the modeling and neurogenesis of neuronal circuits, having implications in the subsequent development of social behavior and cognitive function [149]. An altered gut microbiome leads to the pathogenesis of neurodegenerative diseases by modulating and activating microglial functions. The pathological hallmarks of neurodegenerative diseases are neuroinflammation and microglial activation [163].

Ribosomal 16S RNA sequencing and metagenomics have significantly advanced our understanding of the gut microbiome by allowing the identification of various microorganisms and analysis of their biological pathways. Techniques such as metatranscriptomics and metaproteomics provide a comprehensive view of the microbiome, while culturomics aid in identifying specific microbial species. Various factors, including medications, diet, lifestyle, and genetics, influence the gut microbiome, with medications having a particularly notable impact [164]. Previous research has highlighted the difference between the gut microbiome of patients with ADHD and that of controls [165,166]. In a study evaluating the differences between the gut microbiome of ADHD patients and healthy controls, stool samples from 209 patients were analyzed, and it was found that there was a significant difference in the composition of the fecal microbiome between adult ADHD patients and controls [166].

Another study in which the composition of the gut microbiota was analyzed found that drugs administered to treat ADHD could affect the gut microbiota. The most numerous genus of bacteria associated with ADHD symptoms is the genus Coproccocus [165]. On the other hand, in a study conducted by Aarts and colleagues in 2017, 96 participants were considered, 19 of whom had ADHD and 77 were healthy. The microbiome of people with ADHD was compared to that of healthy individuals by analyzing the 16S microbiome, and the results showed that the genus Bifidobacterium is significantly higher in those with ADHD [157]. Bifidobacterium is one of the many bacteria capable of producing GABA [82]. In addition, in a study conducted by Sukmajaya et al. in 2021, 49 bacterial taxa were differentiated between individuals with ADHD and healthy people; therefore, it is difficult to say which taxa differ the most in ADHD [151].

## 7. Limitations

ADHD can coexist with other mental and physical disorders, such as anxiety, depression, or sleep disorders, and these comorbidities can complicate the assessment of nutrition and oxidative stress in ADHD. Additionally, sleep, physical activity, and other lifestyle factors may interact with food and oxidative stress, making it more difficult to analyze study outcomes. Cultural and regional factors can influence lifestyle and diet, thereby affecting how nutrition and oxidative stress impact ADHD symptoms. Moreover, the studies reviewed vary significantly in terms of the type and duration of nutritional interventions, ranging from ω-3 supplements to specific diets, which may affect the comparability and applicability of the results. However, more research is needed to understand the impact of diet and oxidative stress on ADHD.

## 8. Conclusions

According to our analysis, further research is needed on the diagnosis of ADHD in adults. Although most studies have focused on micronutrient supplementation in children and adolescents, a healthy diet is necessary to reduce ADHD symptoms. A healthy diet refers to eating fruits, vegetables, legumes, and other foods rich in dietary fiber, which play a role in maintaining a balanced microbiota. Dietary approaches, such as the Mediterranean diet and the elimination diet of certain foods that can cause side effects, have also been shown to improve ADHD symptoms. It is especially important to maintain healthy microbiota to prevent the onset of neuropsychiatric disorders.

## Figures and Tables

**Table 1:** Oxidative stress biomarkers from different biological samples and their levels compared to the control.

Sample	Types of Biomarkers	Biomarkers	Level Compared to Control/Treatment	Reference
Plasma	Enzymes	GPx	Significantly lower	[60]
				[63]
			Activity did not change *	[48]
		CAT	Higher than the controls, but this difference was not statistically significant	[60]
			Increased *	[61]
			Significantly lower	[63]
		SOD	Not significantly	[60]
			Significantly lower	[62,63]
	Antioxidants	TAS	Significantly lower	[55]
			Decreased	[68,69]
			No statistical differences	[8]
		GSH	Higher	[9]
		GLA	Significantly higher	[57]
	Oxidants	LPO	Lower *	[61]
		AOPP		
		NOx		
	Enzyme	GRd	Increased *	
	Antioxidant	total thiols	Significantly lower	[8]
	Enzyme	GST	Significantly lower	[63]
Serum	Enzyme	SOD1	Significantly lower	[74]
	Antioxidant	Melatonin	Higher	[59]
		TAC	Significantly lower	[58]
	Enzyme	CAT		
	Antioxidant	GSH		
	Oxidant	MDA	No significant difference between the two groups	
			Increased	[64]
			Statistically significantly Lower	[70]
Saliva	Enzyme	CAT	Reduced	[67]
Rat brain homogenates-Tx MPH	Enzyme	SOD	Decreased *	[71]
			Significantly decreased activity *	[75]
		CAT	Decreased *	[71]
	Antioxidant	GSH	Significantly decreased activity *	[75]
	Enzyme	GPx		
Rat brain homogenates	Antioxidant	GSH	Lower	[73]
	Enzymes	SOD	No difference	
		CAT		
		GPx		
not specified	Oxidant	TOS	Significantly higher	[56]
	Antioxidant	TAS	Lower than in the control group	

*—after treatment MPH; AOPP—advanced oxidation protein product plasma levels; CAT—catalase; GLA—acid γ-linolenic; GPx—glutathione peroxidase; GRd—glutathione reductase activity; GSH—antioxidant glutathione; GST—glutathione-S-transferase; LPO—products of lipid peroxidation; MDA—malondialdehyde; NOx—nitrite + nitrate levels; SOD—superoxide dismutase; TAS—total antioxidant status; SOD1—superoxide dismutase 1; TAC—total antioxidant capacity; TOS—total oxidant status.

**Table 2:** Types of micronutrients and possible effects: focusing on oxidative stress and effects on the brain.

Microutrients	Types	Effects	Food	References
Minerals	Mn	Protects against OS	Grains, rice, nuts, seafood, chocolate, fruits, seeds, leafy green vegetables, teas, juice and water	[100]
	Zn	It helps regulate gene expression, has antioxidant properties and can protect against macular degeneration caused by OS	Meat, seafood, fruits and vegetables, cereals, dairy, legumes, nuts	[101,102]
	Fe	An insufficient iron level can be a risk factor for death	It is found in two forms: heme iron from meat and non-heme iron present in vegetables, legumes, cereals, nuts	[103]
	Mg	It crosses the blood-brain barrier and plays a key role in neuronal maturation and central nervous system function	Vegetables (spinach), pulses, cereals, fruits, nuts	[104,105]
	Se	It acts as a cofactor for GPx enzymes, which play a role in protecting against OS. It reduces lipid oxidation by catalyzing the reduction of peroxides	Vegetables, nuts, fish, grains, meat	[106,107]
Vitamins	B6	Role in neurotransmitter synthesis (gamma-aminobutyric acid, serotonin and dopamine) and stress reduction	Meat, dairy products, beans, nuts, potatoes and more fruits and vegetables	[108,109]
	B12	Essential for DNA function and metabolism. Vitamin B12 deficiency leads to an increase in the proinflammatory cytokine IL-6. Proinflammatory cytokines produce inflammation that increases ROS levels and can lead to OS	Meat, milk, eggs and fish	[110,111]
	D	Role in bone metabolism, brain function and regulates Ca. It can stimulate the activity and expression of GGT, which participates in the glutathione cycle between neurons and astrocytes. It increases the level of glutathione to protect neurons, so it can lead to a decrease in ROS	Sun exposure, fatty fish, dairy products, cereals, orange juice, eggs	[48,112]
Polyunsaturated fatty acids	ω-3 PUFAs	They can prevent chronic diseases, reduce lipoperoxidation levels, and reduce the ratio of SOD/CAT enzymes.High doses of omega-3 fatty acids can trigger OS	Fish (mackerel, salmon), microalgae and some microorganisms	[113,114,115]

Ca—calcium; CAT—catalase; Fe—iron; GGT—gamma-glutamyl transpeptidase; GPx—glutathione peroxidase; IL-6—interleukin-6; Mg—magnesium; OS—oxidative stress; ROS—reactive oxygen species; Se—selenium; SOD—superoxide dismutase; Zn—zinc; ω-3 PUFAs—Omega-3 polyunsaturated fatty acids.

**Table 3:** Micronutrients supplementation in people with ADHD.

Supplements	Number of Participants (*n* =)	Age (Years)	Dose	Time	Result	References
Zn	400	9.61 ± 1.7	150 mg zinc sulfate	12 weeks	Positive effects on the symptoms of ADHD	[141]
	52	6–14	15 mg every morning or two times per day + amphetamine	13 weeks	it had no effect	[142]
	60	9.6 ± 1.70	0.5–1 mg/kg/day methylphenidate + 10 mg Zn	6 weeks	Significantly improved attention	[129]
Fe	23	5–8	80 mg/day ferrous sulfate	12 weeks	Improves ADHD symptoms in children with low serum ferritin levels	[143]
Mg	50	7–12	200 mg/day	6 months	Positive response to Mg supplementation	[144]
Vitamin D	96	9.76 ± 2.38	50,000 UI/week 25-hydroxy-vitamin D3	6 weeks	A positive effect on ADHD symptoms	[145]
	35	7–14	3000 IU/day 25-hydroxy-vitamin D3	12 weeks	may improve cognitive functions related to ADHD	[146]
Mg + Vitamin D	74	6–12	vitamin D (50.000 UI/week) + Mg supplements (6 mg/kg/day)	8 weeks	Reduced social problems and anxiety in children with ADHD	[147]
ω-3 PUFAs	40	8–14	10 g of full fat (80%) margarine daily enriched with either 650 mg of EPA/DHA	16 weeks	ω-3 PUFA supplementation may increase the performance of pharmacological treatments for ADHD	[148]

DHA—docosahexaenoic acid; EPA—eicosapentaenoic acid; Fe—iron; Mg—magnesium; *n*—number of participants; IU—International Units; Zn—zinc; ω-3 PUFAs—Omega-3 polyunsaturated fatty acids.
